# The Performance Analysis Based on SAR Sample Covariance Matrix

**DOI:** 10.3390/s120302766

**Published:** 2012-03-01

**Authors:** Esra Erten

**Affiliations:** Institute of Environmental Engineering, ETH Zurich, Schafmattstr. 6, CH-8093 Zurich, Switzerland; E-Mail: erten@ifu.baug.ethz.ch; Tel.: +41-44-633-4059

**Keywords:** multi-channel systems, sample eigenvalues, Wishart distribution, MIMO, maximum eigenvalue, SAR

## Abstract

Multi-channel systems appear in several fields of application in science. In the Synthetic Aperture Radar (SAR) context, multi-channel systems may refer to different domains, as multi-polarization, multi-interferometric or multi-temporal data, or even a combination of them. Due to the inherent speckle phenomenon present in SAR images, the statistical description of the data is almost mandatory for its utilization. The complex images acquired over natural media present in general zero-mean circular Gaussian characteristics. In this case, second order statistics as the multi-channel covariance matrix fully describe the data. For practical situations however, the covariance matrix has to be estimated using a limited number of samples, and this sample covariance matrix follow the complex Wishart distribution. In this context, the eigendecomposition of the multi-channel covariance matrix has been shown in different areas of high relevance regarding the physical properties of the imaged scene. Specifically, the maximum eigenvalue of the covariance matrix has been frequently used in different applications as target or change detection, estimation of the dominant scattering mechanism in polarimetric data, moving target indication, *etc*. In this paper, the statistical behavior of the maximum eigenvalue derived from the eigendecomposition of the sample multi-channel covariance matrix in terms of multi-channel SAR images is simplified for SAR community. Validation is performed against simulated data and examples of estimation and detection problems using the analytical expressions are as well given.

## 1. Introduction

Multi-channel systems with random nature appear in a wide range of fields in the literature. For several of them, the central limit theorem applies and their random behavior can be modeled by Gaussian statistics, being thus statistically fully described by the first and second order moments. In the case of multi-channel SAR systems, the assumption of zero-mean multivariate complex Gaussian distribution is frequently valid for geophysical media, being thus fully described by the complex Hermitian covariance matrix. This is the statistical case treated in this work.

Since a SAR signal corresponds to the superposition of the scattered fields from all scatters inside a resolution cell, physical parameter estimation may be performed by exploiting the multi-channel covariance matrix. For that, eigendecomposition theorems have been widely used in the literature, decomposing the whole covariance matrix into elementary quantities, in order to provide a better physical interpretation of the data.

The eigendecomposition of the multi-channel SAR covariance matrix leads to useful information in a large range of SAR applications, including Ground Moving Target Indication (GMTI) [1], polarimetric SAR (PolSAR) [2], interferometric SAR (InSAR) [3], polarimetric interferometric SAR (PolInSAR) [4], change detection [5], target detection [6] and filtering [7,8]. Particularly, the covariance matrix maximum eigenvalue has been proved to be a key parameter in various areas. In [5] and [9], the change detection problem was elaborated using the maximum eigenvalue of the interferometric covariance matrix. The eigendecomposition of the SAR covariance matrix with application to SAR GMTI is used in and to calculate the probability of moving target detection in homogeneous and heterogeneous terrain. Regarding polarimetry, the eigendecomposition of the coherency (which is also a covariance) matrix is the most common tool among incoherent target decomposition theorems for the interpretation of the earth’s surface. The maximum eigenvalue of the coherency matrix is also often used in polarimetry, due to the close relationship to the dominant scattering mechanism of the scattering process [2,10,11].

Although there is a wide area of applications regarding the maximum eigenvalue of the multi-channel covariance matrix, a certain lack of information in the literature concerning its statistical characterization in terms of multi-channel SAR may be verified. The statistical description can be useful to understand bias effects and to analyze estimation as well as detection problems. The estimated (sample) covariance matrix follows a Wishart distribution, which has been of interest along the years since its first derivation. In areas as communication systems, several works using such results have been published [12–16]. The application of the Wishart distribution in remote sensing was however considerably late introduced [17].

The cornerstone study considering the statistical description of the covariance matrix eigendecomposition in polarimetry has been carried out in [18]. The majority of the analysis in [18] was performed on the basis of numerical methods. In this paper the results of [18] is supported by addressing analytical solutions. Additionally, it is derived an exact closed form expressions for the Moment Generating Function (MGF) of the sample covariance matrix maximum eigenvalue.

The validation of the derived expressions is performed using simulated data, demonstrating its agreement with the theory. The effect of the number of samples and underlying correlation scenario of the sample covariance matrix on the bias in the estimation of the maximum eigenvalue is also investigated. Finally, examples of applications are as well given, in the fields of estimation and detection theory.

The next section reminds the basics of the statistical description of multi-channel SAR systems and of the covariance matrix eigendecomposition. It also includes the derived theorems presenting the statistical founds. Section 3 includes numerical examples validating the theorems as well as an analysis of their behavior. Sections 4 and 5 show their usage for the analysis of the estimation bias and for the elaboration of detection problems, respectively. Section 6 concludes the paper with discussions and directions for future work. The detailed statistical derivations can be found in the Appendix.

## 2. Statistical Characteristics of the Maximum Eigenvalue of the Multi-Channel Sample Covariance Matrix

### 2.1. Preliminaries

The individual elements of a *m*-dimensional multi-channel system may be organized in a *m*-dimensional vector *k*. As the elements are assumed to follow zero mean complex Gaussian distributions, the vector *k* is said to follow a *m*-dimensional multivariate normal distribution, with zero mean and true covariance matrix Σ among the vector elements, and is represented by $k∼{𝒩}_{m}^{C}(0,\Sigma )$ [19,20]. For zero mean Gaussian statistics the covariance matrix fully describes the data, playing a key role in several application fields. In practical situations however, the true covariance matrix Σ is unknown and has to be estimated by its maximum likelihood estimator (MLE), the sample covariance matrix $Z=(1/n)\sum_{j=1}^{n}k_{j}\;k_{j}{}^{†}$, where *n* is the number of estimation samples and † is the transpose conjugate operator. In the SAR context, the number of independent samples is also called *looks*. The elements of *Z* follow a *m* dimensional complex Wishart distribution with *n* degrees of freedom and true covariance matrix Σ, represented by $Z∼{𝒲}_{m}^{C}(n,\Sigma )$ and defined as [20]:

(1) $$p_{Z}\;(Z)=\frac{n^{\mathrm{mn}}{\left|Z\right|}^{n-m}\;\text{etr}\;(-n\Sigma^{-1}\;Z)}{{\left|\Sigma \right|}^{n}{\tilde{\Gamma }}_{m}\;(n)}\;\;\;\;\;\text{with}\;\;\;\;\;{\tilde{\Gamma }}_{m}\;(n)=\pi^{m(m-1)/2}\;\prod_{i=1}^{m}\Gamma (n-i+1)$$

where Γ(·) is the gamma function and etr(·) is the exponential trace of a matrix.

The spectral theorem from linear algebra allows the decomposition of the full rank *m*-dimensional covariance matrix in a set of *m* one-rank covariance matrices using its eigenvalues and eigenvectors. Accordingly, the decompositions of the true covariance matrix $\Sigma =\sum_{i=1}^{m}\;l_{i}\;(e_{i}\;e_{i}^{†})$ and its estimator $Z=\sum_{i=1}^{m}\;\lambda_{i}\;(e_{i}^{\prime }\;e_{i}^{\prime †})$ are given by

(2) $$\Sigma =Q\left[\begin{array}{ccc} l_{1} & \cdots & 0\\ \vdots & \ddots & \vdots \\ 0 & \cdots & l_{m} \end{array}\right]Q^{†}\;\;\text{and}\;\;Z=Q^{\prime }\left[\begin{array}{ccc} \lambda_{1} & \cdots & 0\\ \vdots & \ddots & \vdots \\ 0 & \cdots & \lambda_{m} \end{array}\right]Q^{\prime †},\;\;\;\begin{array}{c} Q=[e_{1},\;e_{2},\;\cdots ,e_{m}]\\ Q^{\prime }=[e_{1}^{\prime },\;e_{2}^{\prime },\;\cdots ,\;e_{m}^{\prime }] \end{array}$$

with their real non-negative eigenvalues *l_i_* and *λ_i_*, and respective complex eigenvectors *e_i_*, *e*′*_i_*, for *i* = {1, ...*m*}.

### 2.2. Sample Maximum Eigenvalue Statistical Description

The following Theorem III concerns the derived *Moment Generating Function* (MGF) of the maximum eigenvalue of the sample covariance matrix *Z* of a multi-channel statistical system, which is a critical step in removing the bias of the largest eigenvalue. The Theorem III, which is derived due to the previous works (Theorems I and II), is the main result of the paper. It is detailed in the appendix and will be used for the elaboration of illustrative estimation and detection problems in Sections 4 and 5.

Throughout the paper, | · | is the matrix determinant, ${\langle X\rangle }_{n}=(1/n)\sum_{i=1}^{n}\;X_{i}$ denotes the estimator of the random matrix *X* formed from a sample of size *n*.

*Theorem I:* Let $k∼{𝒩}_{m}^{C}(0,\Sigma )$ be a *m*-dimensional complex vector whose elements follow zero mean Gaussian distributions with associated *m* × *m* covariance matrix Σ. Let Σ have *l_m_* ≤ .... ≤ *l*_1_ eigenvalues. Then the *Cumulative Density Function* (CDF) of the maximum eigenvalue *λ_max_* of the sample covariance matrix 〈*kk*^†^〉*_n_*, with the assumption *m* ≤ *n*, is given by

(3) $$F_{\lambda_{\max}}(x)=𝒮|\Psi (x)|,\;\;\;\;\text{with constant term }\;\;𝒮=\frac{\pi^{m(m-1)}n^{m(2n-m+1)/2}}{{\tilde{\Gamma }}_{m}\;(m)\;{\tilde{\Gamma }}_{m}\;(n)}\;\frac{\prod_{k=1}^{m-1}\;k^{m-k}}{\prod_{i=1}^{m}\;l_{i}^{n}\;\prod_{i<j}^{m}\left(\frac{1}{l_{j}}-\frac{1}{l_{i}}\right)}$$

where Ψ(*x*) is a *m* × *m* matrix with its (*i*, *j*)th element $\Psi {\left(x\right)}_{i,j}=\frac{\gamma (n+1-j,x\frac{n}{l_{i}})}{{\left(\frac{n}{l_{i}}\right)}^{\left(n+1-j\right)}}$, and *γ* is the incomplete Gamma function (Equation (2.42) in [21]).

*Proof:* [14] gave the closed expression of CDF of the largest eigenvalue of the complex Wishart matrices. Here, it is written in the form of SAR covariance matrix after small continuation, see also Appendix A.

*Theorem II:* Let $k∼{𝒩}_{m}^{C}(0,\Sigma )$ be a *m*-dimensional complex vector whose elements follow zero mean Gaussian distributions with associated *m* × *m* covariance matrix Σ. Let Σ have *l_m_* ≤ .... ≤ *l*_1_ eigenvalues. Then the *Probability Density Function* (PDF) of the maximum eigenvalue *λ_max_* of the sample covariance matrix 〈*kk*^†^〈*_n_*, with the assumption of *m* ≤ *n*, is given by

(4) $$p_{\lambda \max}\;(x)=𝒮|\Psi (x)|\text{tr}\;\left[\Psi {\left(x\right)}^{-1}\Omega (x)\right]$$

where Ω(*x*) is an *m* × *m* matrix with its *i*, *j*th elements $\Omega {\left(x\right)}_{i,j}=\text{exp}\left(-\frac{n}{l_{i}}x\right)\;x^{n-j}$, and Ψ(*x*) and 𝒮 are defined in Equation (3).

*Proof:* Equation (4) is obtained by differentiating Equation (3) with respect to *x* using (Equation (9) in [22])

(5) $$\frac{d}{\mathrm{dt}}|X(t)|=|X(t)|\;\text{tr}\;\left(X{\left(t\right)}^{-1}\frac{d}{\mathrm{dt}}X(t)\right).$$

Here it can be noted that when the true covariance matrix Σ is diagonal, the PDF of the largest eigenvalue reduces to Ermolaev and Rodyushkin result [23].

*Theorem III:* Let $k∼{𝒩}_{m}^{C}(0,\Sigma )$ be a *m*-dimensional complex vector whose elements follow zero mean Gaussian distributions with associated *m* × *m* covariance matrix Σ. Let Σ have *l_m_* ≤ .... ≤ *l*_1_ eigenvalues. Then for any positive integers *s*, the *s*th moment of the maximum eigenvalue *λ_max_* of the sample covariance matrix 〈*kk*^†^〉*_n_*, with the assumption of *m* ≤ *n*, is given by

(6) $$\begin{array}{ll} E(\lambda_{\max}^{s}) & =𝒮\sum_{i,j=1}^{m}{\left(-1\right)}^{i+j}\sum_{\pi_{s}\in \pi_{\mathrm{sub}}\in S_{m}}\text{sgn}(\pi_{s})\prod_{k=1}^{m-1}\frac{1}{n+1-\pi_{s}(k)}\;{\left(\frac{n}{l_{k}}\right)}^{\frac{n-\pi_{s}(k)}{2}}\;a_{k}^{u_{k}}\;{\left(b+A\right)}^{-v-M}\;\Gamma (v+M)\times \\ & F_{A}\left(v+M;\;u_{1}-\lambda_{1},\ldots ,u_{m-1}-\lambda_{m-1};2u_{1},\ldots ,2u_{m-1};\;\frac{a_{1}}{b_{1}+A},\ldots ,\frac{a_{m-1}}{b_{m-1}+A}\right)\\ & \text{if}\;k=j\wedge \pi_{s}(j)=i,\;\pi_{s}\in \pi_{\mathrm{sub}} \end{array}$$

where 𝒮 indicates the constant term as in *Theorem I* and *F_A_*(· · ·) is the hypergeometric function of several variables with $a_{k}=\frac{n}{l_{k}}$, *u_k_* = (2 + *n* − *π_sub_*(*k*))/2, $v=1+s+n-i+\sum_{k=1}^{m-1}\;\frac{n-\pi_{\mathrm{sub}}\;(k)}{2}$, $b=\frac{n}{l_{j}}+\sum_{k=1}^{m-1}\;\frac{n}{2l_{k}}$, $\lambda_{k}=\frac{n-\pi_{\mathrm{sub}}\;(k)}{2}$, $M=\sum_{k=1}^{m-1}u_{k}$, $A=\frac{1}{2}\sum_{k=1}^{m-1}a_{k}$. Here, the sum is computed over (*m* − 1)! permutations of *π_s_*. *S_m_* denotes the set of all *m*! permutations of the set *S* = {1, 2, ...,*m*}, and sgn(*π_s_*) denotes the sign of the permutation *π_s_* : +1 if *π_s_* is an even permutation and −1 if it is odd.

*Proof:* See Appendix section B. In the appendix the moment of sample maximum eigenvalue is also given in more friendlier form for programming obtained by splitting the hypergeometric function of several variables into a sum of its variables.

### 2.3. Dependence of the Covariance Matrix Eigenvalues

Before starting the following analysis, it would be nice to illustrate the dependencies of the eigenvalues on the covariance matrix parameters, which is necessary for the further understanding of the addressed topics. For that, a two-dimensional covariance matrix is used, originated from the expected values of the outer product of the vector *k* = [*k*_1_ *k*_2_]*^T^*, *i.e.*,

(7) $$\Sigma =E({\mathrm{kk}}^{†})=\left[\begin{array}{cc} \sigma_{1}^{2} & \sigma_{1}\sigma_{2}\rho e^{j\phi }\\ \sigma_{1}\sigma_{2}\rho e^{-j\phi } & \sigma_{2}^{2} \end{array}\right]$$

where $\sigma_{1}^{2}$, $\sigma_{2}^{2}$ are the variance or power of *k*_1_ and *k*_2_, respectively, *ρ* is the absolute and *ϕ* the phase of their complex correlation coefficient, and *T* means transpose.

The eigenvalues of the true covariance matrix can be shown to be given by [5]:

(8) $$l_{1,2}=\frac{1}{2}\left[\frac{b+1}{\sqrt{b}}\pm \sqrt{\frac{b^{2}+1}{b}-2+4\rho^{2}}\right],\;\;\;\;\sigma_{1}^{2}=b\sigma_{2}^{2}.$$

**Figure 1.**
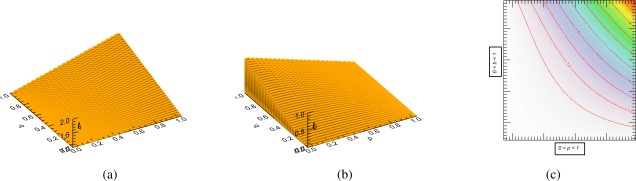
Two-dimensional system configuration. (**a**) True eigenvalue *l*_1_ and (**b**) true eigenvalue *l*_2_ as a function of correlation and normalized power ratio *b*. (**c**) Counter plots of *l*_1_.

The behavior of *l*_1_ and *l*_2_ is presented in Figure 1, as a function of a normalized power ratio *b* and a correlation *ρ*. Note their mutual dependence, making clear that *b* and *ρ* directly determine the behavior of both eigenvalues.

## 3. Validation and Analysis of the Theoretical Expressions

This section aims to validate the theorems mentioned in the previous section using simulated data. The simulated data have been generated using different multi-dimensional configurations, where the correlation between channels have been generated using the well known Mahalanobis transformation [24].

**Figure 2.**
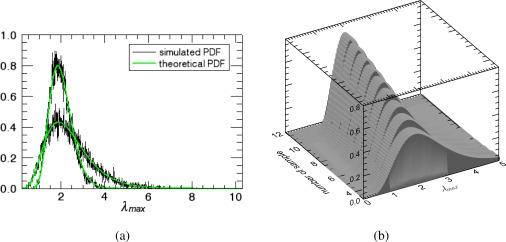
(**a**) Comparison between the theoretical distributions and the histograms obtained from simulated data of the maximum sample eigenvalue. (**b**) The distribution of the maximum sample eigenvalue as a function of the number of samples, for a three-dimensional system. In both case, the powers are given by *σ*_*k*_1__ = *σ*_*k*_2__ = *σ*_*k*_3__ = 1 and correlations by *ρ*_*k*_1_*k*_2__ = 0, *ρ*_*k*_1_*k*_3__ = 0.8 and *ρ*_*k*_2_*k*_3__ = 0. When *n* → ∞, *λ_max_* = *l*_1_ = 1.8.

Figure 2(a) shows the comparison of the Equation (4) with simulations. The theoretical PDF curves clearly agree with the histograms obtained from simulated data. As expected, the PDFs become narrower with increasing *n*, indicating less variance around the true value of the maximum eigenvalue *l*_1_ = 1.8. This behavior can be better seen in Figure 2(b) where the distribution of *λ_max_* as a function of the number of estimation samples *n* is presented. Note also that the expected value of the distributions seems to change for different *n*.

**Figure 3.**
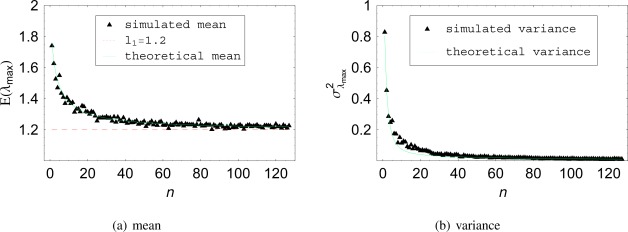
Theoretical *versus* simulation results (**a**) for the first order statistics (mean), and (**b**) for the second order statistics (variance), of the sample maximum eigenvalue of a two-dimensional system with powers *σ*_*k*_1__ = *σ*_*k*_2__ = 1 and correlation *ρ*_*k*_1_*k*_2__ = 0.2.

Figure 3(a) shows the variation of the histogram mean as a function of *n* for a two-dimensional case with fix correlation *ρ* = 0.2. In Figure 3(a), the theoretical expected value of *λ_max_* has been also over plotted for comparison. The curves match well, which validates *Theorem III*. The same has been carried out for the second order moment, *i.e.*, the variance of *λ_max_*, and is presented in Figure 3(b). The agreement between the theoretical variance of *λ_max_* and the variance of the simulated data can again be confirmed. Also, as already observed in Figure 2(a), the lower the number of samples *n*, the higher the variance of *λ_max_*.

**Figure 4.**
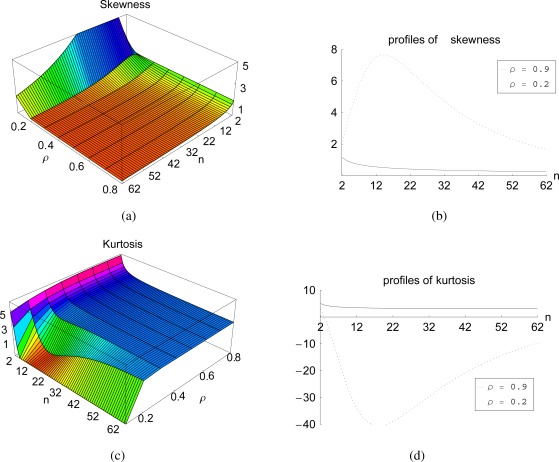
Theoretical results for the third (skewness) and the fourth (kurtosis) order statistics of the sample maximum eigenvalue of a 2D system having powers *σ*_*k*_1__ = *σ*_*k*_2__ = 1 and correlations *ρ* = {0.2, 0.3, . . ., 0.9} versus the number of samples *n* = {2, 3, . . ., 62}.

The impact of the correlation and the number of samples on the behavior of the third (skewness) and fourth (kurtosis) order moment of the maximum sample eigenvalue *λ_max_* is presented in Figure 4. Skewness is a measure of how symmetrical the distribution is with respect to its mean. Figure 4(a,b) indicates that the skewness of *λ_max_* converges to zero for increasing *n* and increasing *ρ*, expressing the tendency to a symmetrical distribution in that cases.

Kurtosis is a measure of the peakedness of the distribution. Figure 4(c,d) shows that for increasing *n* and *ρ*, the kurtosis of the *λ_max_* distribution tends to three, which corresponds to the kurtosis of the normal Gaussian distribution.

## 4. Estimation Bias

**Figure 5.**
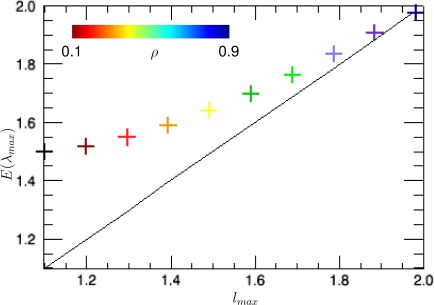
Effects of the correlation between channels on the expected value of the maximum sample eigenvalue keeping *n* = 3 and *k* = 1.

Figure 5 shows the expected value (first order moment) of the maximum sample eigenvalue *λ_max_* as a function of the true eigenvalues *l_max_* for a fixed number of samples *n* = 3 and *k* = 1. The variation of the underlying correlation of the true covariance matrix *ρ*, which changes the values of *l_max_*, has been as well indicated in a color code. Observe that the higher the correlation is, the higher *l_max_* becomes.

The *bias* of the estimator of a certain parameter is defined as the difference between the expected value of the estimator and the true value of the parameter. In the present case, the bias is hence given by *E*[*λ_max_*] − *l_max_*. Notice thus from Figure 5 that the bias of *λ_max_* becomes very strong for low values of the correlation *l_max_* (or equivalently, low values of *ρ*).

**Figure 6.**
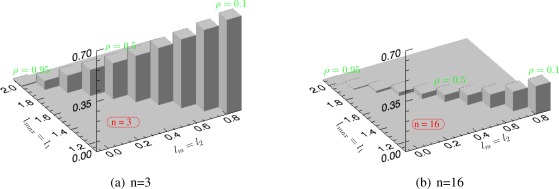
The bias, *E*[*λ_max_*] − *l_max_*, of the sample maximum eigenvalue with the number of samples 3 and 16 in various correlated channels having a standard deviation of *σ*_*k*_1__ = *σ*_*k*_2__ = 1.

The effect is emphasized in Figure 6(a,b), where the bias of *λ_max_* is presented as a function of *l_max_* and *l*_2_, for *n* = 3 and *n* = 16, respectively. The values of the underlying correlation *ρ* have been also indicated. The bias is small either when the correlation is high or the number of samples is sufficiently large. When the correlation between channels is low, the bias becomes very significant and a large number of samples is necessary to decrease the estimation bias.

## 5. Analysis of Detection Problems

Detection theory is a means to quantify the ability of a procedure to detect a parameter (or signal) immersed in a noise environment. A decision has to be taken, in order to say yes, there is a signal, or no, there is no signal. Such decision is usually taken under the application of a decision threshold. Noise contributes off course negatively inducing wrong decisions. For the quantification of detection accuracy, some quantities are usually defined as the probability of detection (*PD*) and probability of false alarm (*PFA*), allowing a detection problem analysis.

The maximum eigenvalue of covariance matrices has been frequently used in the literature for the elaboration of certain detection problems. In the Ground Moving Target Indication (GMTI) area, for instance, the signal to clutter plus noise ratio has been studied under the distribution of the sample eigenvalues, which allowed the implementation of a constant false alarm rate detector [9]. The eigenvalues of the covariance matrix of a SAR image pair have been also used in order to formulate a problem in the change detection area [5]. Another example is the determination of the existence of just a single dominant scattering mechanism in a SAR polarimetric acquisition, which also has been evaluated using a threshold in the maximum sample eigenvalue of the polarimetric covariance matrix [11]. Target detection and polarimetric filtering represent other fields of application in which the maximum eigenvalue can be used.

Having the closed form expressions of the PDF (Equation (4)) and/or CDF (Equation (3)) of the maximum sample eigenvalue of the covariance matrix, the *PD* and *PFA* when applying a threshold in *λ_max_* can be analytically computed, allowing a complete detection problem analysis.

### 5.1. Detection of a Dominant Maximum Eigenvalue

Since several detection problems rely in fact on the choice of a dominant or non-dominant *l_max_* by applying a threshold in *λ_max_*, the following problem with two hypotheses is elaborated

$$\begin{array}{c} H_{0}\;(l_{\max}\;\text{is dominant}):l_{\max}=l_{1}\;\;\;\text{and }\;\;l_{i}=0\\ H_{1}\;(l_{\max}\;\text{is not dominant}):l_{\max}=l_{1}=l_{i} \end{array}$$

for *i* = {2, 3, . . ., *m*}.

In one application area *H*_0_ may mean that just a single scattering mechanism is present inside a resolution cell of polarimetric SAR data, in other application *H*_0_ can mean that a change happened or a moving target is present in the scene.

Since *l_max_* is not achievable, a threshold 𝒯 has to be used in *λ_max_*, originating the following *PD* and *PFA*

$$\begin{array}{lll} \mathrm{PD} & = & p[\text{accepting }H_{0}|H_{0}\;\text{is true}]\\ & = & p[\lambda_{\max}>𝒯|H_{0}\;\text{is true}]=\int_{𝒯}^{\infty }p_{\lambda_{\max}}(x;\;H_{0})\;dx\\ \mathrm{PFA} & = & p[\text{accepting }H_{0}|H_{1}\;\text{is true}]\\ & = & p[\lambda_{\max}>𝒯|H_{1}\;\text{is true}]=\int_{𝒯}^{\infty }p_{\lambda_{\max}}(x,\;H_{1})\;dx \end{array}$$

as a function of the decision threshold 𝒯.

Hence, for a two-dimensional system configuration, the distribution p_*λ*_*max*__ (*x; H*_0_) is the distribution of *λ_max_* when *l*_1_ = 2 and *l*_2_ = 0. For a three-dimensional system, p_*λ*_*max*__ (*x; H*_0_) is determined evaluating the distribution of *λ_max_* when *l*_1_ = 3 and *l*_2_ = *l*_3_ = 0. Higher dimensional system configurations follow the same rule.

On the other hand, the distribution p_*λ*_*max*__ (*x; H*_1_) is given by *l*_1_ = *l*_2_ = 1 and *l*_1_ = *l*_2_ = *l*_3_ = 1 for a two- and three-dimensional system, respectively.

For each value of 𝒯, there exists a pair (*PFA, PD*). The curves of *PD* versus *PFA* are called Receiver Operating Characteristic (ROC) curves and express the detection performance. The more a ROC curve bends toward the upper left, the better is the detection performance since a higher *PD* and lower *PFA* is achieved.

**Figure 7.**
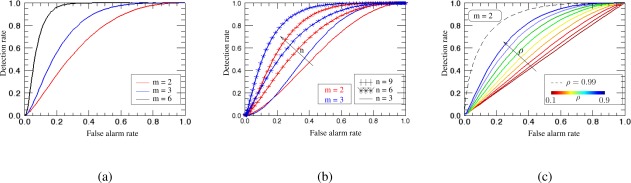
Maximum sample eigenvalue detection performance as a function of the number or channels *m*, samples *n* and correlation *ρ*. (**a**) ROC curves for different multidimensional systems, *m* = {2, 3, 6} and *n* = 6. (**b**) ROC curves for the two- and three-dimensional cases, and for different number of samples *n*. (**c**) ROC curves for the two-dimensional case, *n* = 3, and for different correlation.

Figure 7(a) shows the detection performance as a function of the dimension of the multichannel system, *i.e.*, *m* = 2, e.g., interferometric, *m* = 3, e.g., polarimetric and *m* = 6, e.g., polarimetric-interferometric system. For all cases, with fixed number of samples *n* = 6, one can realize that the performance of detection is significantly improved as the number of SAR images increases. Figure 7(b) shows the ROC curves for *m* = {2, 3} and for different number of samples *n*. It can be seen that for both multidimensional system configurations the number of samples increases the detection performance.

In order to state how correlation effects the detection performance, the detection problem is reformulated as follows

$$\begin{array}{c} H_{0}\;(l_{\max}\;\text{is dominant}):l_{\max}=l_{1}>l_{2}\geq l_{3},\ldots ,\geq l_{m}\geq 0\\ H_{1}\;(l_{\max}\;\text{is not dominant}):l_{\max}=l_{1}=l_{i}\;i=\{2,3,\ldots ,m\}. \end{array}$$

In this way, every time that correlation is greater than zero the eigenvalues have different values, and one is larger than the others. For the two-dimensional case, for instance, p_*λ*_*max*__ (*x; H*_0_) is the distribution of *λ_max_* when *l*_1_ > *l*_2_, which changes for different values of the correlation *ρ*. Figure 7(c) presents the ROC curves for this case. For small correlation between channels, the detector suffers from a significant false-alarm rate. As expected, when correlation increases the ROC curve bends toward the upper left, indicating better detection performance. The limit is reached when *ρ* = 1, meaning that *l*_1_ = 2 and *l*_2_ = 0, which corresponds to the cases of the previous detection problem.

### 5.2. Target Detection Using Polarimetric Matched Filter

In this section the application of the expressions for the detection of specific targets using the Polarimetric Matched Filter (PMF) concept is illustrated. For that, a short review on PMF is required [6,7].

In a polarimetric acquisition, the dimension of the system corresponds to the number of channels, which are in general four, but three for the backscattering reciprocal case. The measured vector *k* is hence three- or four-dimensional. The elements of the vector *k* may be weighted in a given way, in order to satisfy a certain condition. The weighting can be accounted for by making *y* = *h*^†^*k*, where *h* is a complex vector with same dimension as *k*. A condition usually aimed to be satisfied in the literature is the maximization of the quadratic detector |*y*|^2^ (Equation (59) in [6]). The optimal weighting vector *h* in order to detect a distributed target with the target vector *k* is dependent on the target characteristics. Accordingly, the expected value of |*y*|^2^ is given by

(9) $$E\{{\left|y\right|}^{2}\}=E\{h^{†}k{\left(h^{†}k\right)}^{†}\}=h^{†}\Sigma_{t}\;h$$

where Σ*_t_* = *E*{*kk*^†^} is the target covariance matrix. Regarding Rayleigh quotient (Theorem 15.91 in [25]), for any *m*-dimensional complex vector *x* and a given *m* × *m* Hermitian matrix *A*, *x*^†^*Ax* ≤ ||*x*||^2^*a_max_*, where *a_max_* is the maximum eigenvalue of *A*. The equality is valid if *x* is along the direction of the *a_max_* eigenvector *U_max_* (||*U_max_*|| = 1). Hence, for a given distributed target with covariance matrix Σ*_t_*, the optimum weighting vector *h* is given by the eigenvector of the maximum eigenvalue of Σ*_t_*.

The target vector *k* is a random sample and has alone no physical meaning. Therefore, usually multi-look processing is performed making, for *n* samples (or looks)

(10) $${\bar{y}}^{2}=\sum_{i=1}^{n}{\left|h^{†}k_{i}\right|}^{2}.$$

The target is assumed to be immersed in polarization independent clutter. In this way, the detection procedure is thus evaluated by choosing *h* as the eigenvector corresponding to the maximum eigenvalue of the covariance matrix Σ*_t_* of the target to be detected, and applying a detection threshold *y̅*^2^ > 𝒯.

In the presence of target *y̅*^2^ is maximum, given by *y̅*^2^ = ||*h̅*||^2^*λ_max_*, where *h̅* is the eigenvector of *λ_max_*. Hence, assuming without loss of generality that *h̅* has unitary length (||*h̅*||^2^ = 1), detection performances can be made using the derivations given in this work, as done in the previous section, where a threshold has been used in order to detect a dominant maximum sample eigenvalue (*λ_max_* = *y̅*^2^ > 𝒯).

Note that the probabilities of detection and false alarm defined in last section can be more simply evaluated by

(11) $$\begin{array}{lll} \mathrm{PD} & = & \int_{𝒯}^{\infty }p_{\lambda_{\max}}(x;\;H_{0})\;dx=1-\int_{-\infty }^{𝒯}p_{\lambda_{\max}}(x;\;H_{0})\;dx=1-F_{\lambda_{\max}}(𝒯;\;H_{0})\\ \mathrm{PFA} & = & 1-F_{\lambda_{\max}}(𝒯;\;H_{1}) \end{array}.$$

**Figure 8.**
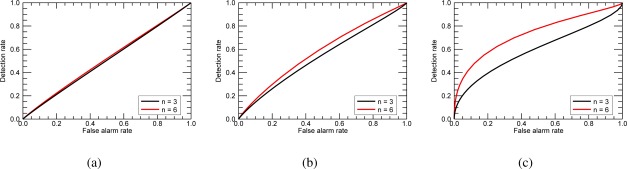
Detection performance of three different distributed scatterers using the PMF concept for different number of samples. (**a**) Azimuthal symmetric scatterer with *ɛ* = 1, *γ* = 0.5 and *ρ* = 0. (**b**) Reflection symmetric scatterer with *ɛ* = 1, *γ* = 0.8 and *ρ* = 0.8. (**c**) Reflection symmetric scatterer with *ɛ* = 0, *γ* = 0.8 and *ρ* = 0.8. The *PFA* is evaluated using the polarization independent clutter with *ɛ* = 1, *γ* = 1 and *ρ* = 0.

A three-dimensional polarimetric target detection problem was formulated making *k* = [*S_HH_* *S_HV_* *S_VV_*]*^T^*, where *S_i_* is the polarimetric scattering matrix element in channel *i*, and using the target covariance matrix structure

(12) $$\Sigma_{t}=S_{HH}\left[\begin{array}{ccc} 1 & 0 & \rho \sqrt{\gamma }\\ 0 & 2ɛ & 0\\ \rho *\sqrt{\gamma } & 0 & \gamma \end{array}\right].$$

where $ɛ=\frac{E\{{\left|S_{\mathrm{HV}}\right|}^{2}\}}{E\{{\left|S_{\mathrm{HH}}\right|}^{2}\}}$, $\gamma =\frac{E\{{\left|S_{\mathrm{VV}}\right|}^{2}\}}{E\{{\left|S_{\mathrm{HH}}\right|}^{2}\}}$ and *ρ* is the complex correlation coefficient between *S_HH_* and *S_VV_* [6,26]. Figure 8 shows the ROC curves for different number of samples for three different kinds of targets. Figure 8(a) corresponds to an azimuthal symmetric target having covariance matrix with parameters *ɛ* = 1, *γ* = 0.5 and *ρ* = 0. Figure 8(c) corresponds to a reflection symmetric target having covariance matrix with parameters *ɛ* = 1, *γ* = 0.8 and *ρ* = 0.8, while Figure 8(b) to a reflection symmetric target with parameters *ɛ* = 0, *γ* = 0.8 and *ρ* = 0.8 in its covariance matrix. For all three cases, *S_HH_* = 1 and the *PFA* was evaluated using the polarization independent clutter having *ɛ* = 1, *γ* = 1 and *ρ* = 0. Note that the curves vary not just with the number of used samples *n* but are also different for different targets having different Σ*_t_*. This means that some types of targets are easier to detect than others, when using the quadratic detector described here and when the clutter is polarization independent. When the target covariance matrix is similar to the one of the clutter, the detection performance weakens (Figure 8(a)). On the other hand, when the covariance matrix of the target is significantly different from the clutter one, the detection performance improves (Figure 8(c)).

## 6. Conclusions and Discussion

In this paper a depth statistical analysis of the maximum eigenvalue of the eigendecomposition of the sample covariance matrix in terms of SAR applications is presented. The proposed analysis are supported by simulation results via several examples. The results are based on a exact closed-form expressions of PDF, CDF and MGF. In this study, existing density functions of the sample maximum eigenvalue were extended and/or implemented into multi-channel SAR system in order to obtain a simple expression of the sample eigenvalues giving a way to fruitful applications. From these closed-form expressions, it has been possible to develop new algorithms for unbiased calculations of parameters extracted from multi-channel SAR covariance matrix. In addition to these implementations, closed-form expressions were developed for the MGF of the sample maximum eigenvalue, which can be critical in the area of bias removal and detection performance analysis. This new closed-form expressions of the MGF can be also interesting for other application areas like MIMO systems (Multiple-Input Multiple-Output). Apart from estimation theory analysis including the MGF, the detection problem of the sample maximum eigenvalue has been also discussed.

## Appendix

This Appendix starts with the reminder of some basic results from linear algebra and statistical theory to prove the Theorem III proposed in this manuscript.

*Definition 1* (Theorem A3 in [19]): The determinant of a square *m* × *m* matrix *A* is

(A1) $$|A|=\sum_{\pi }\text{sgn}(\pi )a_{1k_{1}}\;a_{2k_{2}\cdots }a_{mk_{m}}=\sum_{\pi }\text{sgn}(\pi )\prod_{i=1}^{m}a_{ik_{i}}$$

where ∑*_π_* denotes the summation over all *m*! permutations, and sgn(*π*) denotes the sign of the permutations determined by (−1)*^π^*, where *π* is the permutation symbol. Notice that the determinant is a combination of the determinants of its sub-matrices, and permuting the columns or rows of a matrix changes the sign of the determinant related to the permutation.

*Definition 2* (Corollary 2 in [27]): Given two arbitrary *m* × *m* matrices Φ(*x*) and Ψ(*x*) with *ij*th elements Φ*_i_*(*x_j_*) and Ψ*_i_*(*x_j_*), and an arbitrary function *ξ*(·), the following identity holds:

(A2) $${\int \cdots \int_{𝒟}|\Phi (x)|\;|\Psi (x)|\prod_{k=1}^{m}\xi (x_{k})dx_{1}dx_{2}\ldots dx_{m}=|\left\{\int_{a}^{b}\Phi_{i}(x)\Psi_{j}(x)\xi (x)dx\right\}\|}_{i,j=1}^{m}$$

where the multiple integral is over the domain 𝒟 = {*a* ≤ *x_m_* ≤ · · · ≤ *x*_2_ ≤ *x*_1_ ≤ *b*}.

Proof of Theorem I

In the case *m* ≤ *n*, the sample covariance matrix has full rank and follows a central Wishart distribution. It can be noted that [28] is an interesting work concerning the case *n* ≤ *m*. In Wishart history, the joint PDF of the real ordered sample eigenvalues ∞ ≥ *λ*_1_ ≥ . . . ≥ *λ_m_* ≥ 0 of *Z* is given by Equation (36) in [18]

(A3) $$\begin{array}{lll} p_{\Xi }(\Xi ) & = & \frac{\pi^{m(m-1)}\;n^{m(2n-m+1)/2}}{{\tilde{\Gamma }}_{m}\;(m)\;{\tilde{\Gamma }}_{m}\;(n)}|{\text{exp}\left(-n\frac{\lambda_{j}}{l_{i}}\right)\|}_{i,j=1}^{m}\prod_{k=1}^{m-1}k^{m-k}\frac{\prod_{i=1}^{m}\;\lambda_{i}^{n-m}\;\prod_{i<j}^{m}\left(\lambda_{i}-\lambda_{j}\right)}{\prod_{i=1}^{m}\;l_{i}^{n}\;\prod_{i<j}^{m}\;\left(\frac{1}{l_{j}}-\frac{1}{l_{i}}\right)},\\ & & \text{with}\;\Xi =Q^{H}{\langle Z\rangle }_{n}Q \end{array}$$

where Ξ = diag{*λ*_1_, . . ., *λ_m_*}, p_Ξ_(Ξ) is the joint PDF of the eigenvalues of the sample covariance matrix ∞ ≥ *λ*_1_ ≥ . . . ≥ *λ*_*m*−1_ ≥ *λ_m_* ≥ 0 and ∞ ≥ *l*_1_ ≥ ... ≥ *l*_*m*−1_ ≥ *l_m_* ≥ 0 are the eigenvalues of the true covariance matrix.

The CDF of the maximum eigenvalue *λ_max_* = *λ*_1_ is obtained using

(A4) $$\begin{array}{lll} F_{\lambda_{\max}}\;(x) & = & p(\lambda_{\max}\leq x)\\ & = & p(\lambda_{m}\leq x,\ldots ,\lambda_{1}\leq x)\\ & = & \int_{𝒟}p(\lambda_{1},\;\lambda_{2},\ldots ,\lambda_{m})d\lambda_{1}\ldots d\lambda_{m} \end{array}$$

where *p*(*λ*_1_, *λ*_2_, ..., *λ_m_*) is the joint PDF of the eigenvalues as defined in Equation (A3), and 𝒟 = {0 ≤ *λ_m_* ≤ ... ≤ *λ*_1_ ≤ *x*}. Before substituting Equation (A3) in Equation (A4) it is desirable to write a friendlier expression for the joint PDF Equation (A3). If the constant is denoted by 𝒮 as in Equation (3), then Equation (A3) can be written as

(A5) $$p_{\Xi }(\Xi )=𝒮\prod_{i=1}^{m}\lambda_{i}^{n-m}\prod_{i<j}^{m}\left(\lambda_{i}-\lambda_{j}\right)|{\text{exp}\left(-n\frac{\lambda_{j}}{l_{i}}\right)\|}_{i,j=1}^{m}.$$

Due to the definition of the Vandermonde determinant [25] the multiplication of $\prod_{i=1}^{m}\lambda_{i}^{n-m}$ and $\prod_{i<j}^{m}\left(\lambda_{i}-\lambda_{j}\right)$ is:

(A6) $$\left[\begin{array}{ccc} \lambda_{1}^{n-m} & 0 & 0\\ 0 & \ddots & 0\\ 0 & 0 & \lambda_{m}^{n-m} \end{array}\right]\cdot \left[\begin{array}{ccc} \lambda_{1}^{m-1} & \cdots & \lambda_{1}^{m-m}\\ \vdots & \ddots & \cdots \\ \lambda_{m}^{m-1} & \cdots & \lambda_{m}^{m-m} \end{array}\right]=\left[\begin{array}{ccc} \lambda_{1}^{n-1} & \cdots & \lambda_{1}^{n-m}\\ \vdots & \ddots & \cdots \\ \lambda_{m}^{n-1} & \cdots & \lambda_{m}^{n-m} \end{array}\right]\to |\lambda_{i}^{n-j}|{|}_{i,j=1}^{m}$$

Then the joint PDF of the sample eigenvalues can be written in the form of

(A7) $$p_{\Xi }(\Xi )=𝒮|\lambda_{i}^{n-j}|{|}_{i,j=1}^{m}\cdot {|\text{exp}\left(-n\frac{\lambda_{j}}{l_{i}}\right)\|}_{i,j=1}^{m}$$

By applying the result of *Definition 2* and *Definition 1* into (A4) with $\Psi_{i}(\lambda_{j})=\lambda_{i}^{n-j}$ and $\Phi_{i}(\lambda_{j})=\text{exp}\left(-n\frac{\lambda_{j}}{l_{i}}\right)$, it yields

(A8) $$F_{\lambda_{\max}}(x)=𝒮\left|\int_{0}^{x}\lambda^{n-j}\text{exp}(-n\frac{\lambda }{l_{i}})d\lambda \right|.$$

Finally, solving the remaining integral using Equation (351) in [25] yields the desired closed-form expression of the sample maximum eigenvalue CDF of multi-channel covariance matrix

(A9) $$F_{\lambda_{\max}}(x)=𝒮|\Psi {\left(x\right)}_{i,j}|\;\text{with }\Psi {\left(x\right)}_{i,j}={\left(\frac{n}{l_{i}}\right)}^{\left(j-n-1\right)}\gamma (n+1-j,x\frac{n}{l_{i}})$$

where $\gamma (a,x)=\int_{0}^{x}x^{a-1}e^{-x}dx$ denotes the lower incomplete gamma function.

Proof of Moments of the Sample Maximum Eigenvalue

For any positive integer *s*, the *s*th moment of the maximum eigenvalue is obtained using

(A10) $$E(\lambda_{\max}^{s})=\int_{0}^{\infty }x^{s}p_{\lambda_{\max}}\;(x)dx$$

where p_*λ*_*max*__ (*x*) is the PDF of *λ_max_*. It has been shown in Equation (4) that

(A11) $$p_{\lambda_{\max}}(x)=𝒮|\Psi (x)|\text{tr}\;\left[\Psi {\left(x\right)}^{-1}\Omega (x)\right]$$

where Ω(*x*) and Ψ(*x*) are *m* × *m* matrices with *ij*th element $\Omega {\left(x\right)}_{i,j}=\text{exp}\left(-\frac{n}{l_{i}}x\right)\;x^{n-j}$ and $\Psi {\left(x\right)}_{i,j}={\left(\frac{n}{l_{i}}\right)}^{-(n+1-j)}\gamma (n+1-j,\;x\frac{n}{l_{i}})$, and 𝒮 denotes the constant terms given in Equation (3).

It is desirable to write a friendlier expression for Equation (A11) to be useful for deriving the moments of the sample maximum eigenvalue. Due to the definition of the inverse of the *m* × *m* matrix $A^{-1}=C_{\mathrm{ij}}^{T}/|A|$, Equation (A11) can be written in the following form

(A12) $$p_{\lambda_{\max}}(x)=𝒮\;\text{tr }[C^{T}\;(x)\Omega (x)]$$

where *C_T_* (*x*) is the transpose of the cofactor matrix Ψ(*x*). Since the trace of a square matrix is the sum of the diagonal elements of the matrix, Equation (A11) can be written as the sum of the diagonal elements of *D*(*x*) = (*C^T^* (*x*)Ω(*x*)).

(A13) $$E(\lambda_{\max}^{s})=𝒮\int_{0}^{\infty }x^{s}\text{tr }[C^{T}\;(x)\Omega (x)]dx$$

(A14) $$=𝒮\int_{0}^{\infty }x^{s}\text{tr }[D]dx$$

Now the problem in Equation (A11) is the evaluation of the diagonal elements of the *D*(*x*). The matrix *D*(*x*) is the product of the two square matrices and the trace of the product of two matrices is $\text{tr}(C^{T}\Omega )=\sum_{i,j=1}^{m}C_{\mathrm{ji}}\;\Omega_{\mathrm{ji}}=C_{11}\;\Omega_{11}+\cdots +C_{m1}\;\Omega_{m1}+\cdots +C_{1m}\;\Omega_{1m}+\cdots +C_{\mathrm{mm}}\;\Omega_{\mathrm{mm}}$ shown as

(A15) $$\mathrm{tr}\;\left(\left[\begin{array}{cccc} C_{11} & C_{21} & \cdots & C_{m1}\\ C_{12} & C_{22} & \cdots & C_{m2}\\ \vdots & \vdots & \ddots & \vdots \\ C_{1m} & C_{2m} & \cdots & C_{\mathrm{mm}} \end{array}\right]\cdot \left[\begin{array}{cccc} e^{-x\frac{n}{l_{1}}}\;x^{n-1} & e^{-x\frac{n}{l_{1}}}\;x^{n-2} & \cdots & e^{-x\frac{n}{l_{1}}}\;x^{n-1}\\ e^{-x\frac{n}{l_{2}}}\;x^{n-1} & e^{-x\frac{n}{l_{2}}}\;x^{n-2} & \cdots & e^{-x\frac{n}{l_{2}}}\;x^{n-2}\\ \vdots & \vdots & \ddots & \vdots \\ e^{-x\frac{n}{l_{m}}}\;x^{n-1} & e^{-x\frac{n}{l_{m}}}\;x^{n-2} & \cdots & e^{-x\frac{n}{l_{m}}}\;x^{n-m} \end{array}\right]\right)$$

where *C_ji_* is the determinant of the sub-matrix *M_ji_* obtained from Ψ(*x*) by removing its *j*th row and *i*th column. Then, the *j*, *i* th cofactor of Ψ*_ji_*, denoted by *C_ji_*, is *C_ji_* = (−1)^*i*+*j*^ |*M_ji_*|. Now, Equation (A14) is:

(A16) $$E(\lambda_{\max}^{s})=𝒮\sum_{j,i=1}^{m}\int_{0}^{\infty }x^{s}\;C_{\mathrm{ji}}\;\Omega_{\mathrm{ji}}\;dx$$

where *C_ji_* can be extracted from Ψ(*x*) by *Definition I* as follows,

(A17) $$C_{\mathrm{ji}}=\frac{{\left(-1\right)}^{i+j}\sum_{\pi_{\mathrm{sub}}\in S_{m}}\text{sgn}(\pi_{\mathrm{sub}})\prod_{k=1}^{m}\Psi_{k,\pi_{\mathrm{sub}}(k)}}{\Psi_{j,i}},\;\text{if }k=j\wedge \pi_{s}(j)=i,\pi_{s}\in \pi_{\mathrm{sub}}.$$

Here, the sum is computed over (*m* − 1)! permutations of *π_s_*. *S_m_* denotes the set of all *m*! permutations of the set *S* = {1, 2, ...,*m*}, and sgn(*π_sub_*) denotes the sign of the permutation *π_s_* : +1 if *π_s_* is an even permutation and −1 if it is odd. After interchanging the order of the summations and integral in Equation (A16), the integration reduces to the following form

(A18) $$\begin{array}{ll} E(\lambda_{\max}^{s}) & =𝒮\sum_{i,j=1}^{m}{\left(-1\right)}^{i+j}\int_{0}^{\infty }x^{s}\;\text{exp}\left(-\frac{n}{l_{j}}x\right)\;x^{n-i}\\ & \times \left(\frac{\sum_{\pi_{\mathrm{sub}}\in S_{m}}\text{sgn}(\pi_{\mathrm{sub}})\prod_{k=1}^{m}\gamma (n+1-\pi_{\mathrm{sub}}(k),\;x\frac{n}{l_{k}})\;{\left(\frac{n}{l_{k}}\right)}^{\pi_{\mathrm{sub}}(k)-n-1}}{\gamma (n+1-i,\;x\frac{n}{l_{j}})\;{\left(\frac{n}{l_{j}}\right)}^{i-1-n}}\right)\;dx\\ & \Rightarrow 𝒮\sum_{i,j=1}^{m}{\left(-1\right)}^{i+j}\sum_{\pi_{\mathrm{sub}}\in S_{m}}\text{sgn}(\pi_{\mathrm{sub}})\int_{0}^{\infty }x^{s}\text{exp}\left(-\frac{n}{l_{j}}x\right)\;x^{n-i}\\ & \times \left(\frac{\prod_{k=1}^{m}\gamma (n+1-\pi_{\mathrm{sub}}(k),\;x\frac{n}{l_{k}})\;{\left(\frac{n}{l_{k}}\right)}^{\pi_{\mathrm{sub}}(k)-n-1}}{\gamma (n+1-i,\;x\frac{n}{l_{j}})\;{\left(\frac{n}{l_{j}}\right)}^{i-1-n}}\right)dx. \end{array}$$

It can be seen from Equation (A18) that the integration includes the multiplication of (*m* − 1) times incomplete gamma functions with exponential and powers. After applying identities (ex. 10.3, Equations 10.15 and 10.65 in [21]),

(A19) $$\begin{array}{c} \gamma (n,\;\mathrm{ax})=\frac{1}{n}\;{\left(\mathrm{ax}\right)}^{\frac{n-1}{2}}\;e^{\frac{-\mathrm{ax}}{2}}\;{𝒨}_{\frac{n-1}{2},\;\frac{n}{2}}\;(\mathrm{ax}),\;\frac{n}{2}\neq \end{array}-1,\;-2,\;-3,\ldots ,$$

(A20) $$\gamma \left(n+1-\pi_{\mathrm{sub}}(k),\;\frac{n}{l_{k}}\;x\right)=\frac{1}{n+1-\pi_{\mathrm{sub}}(k)}\;{\left(\frac{n}{l_{k}}x\right)}^{\frac{n-\pi_{\mathrm{sub}}(k)}{2}}\;e^{\frac{-\mathrm{nx}}{2l_{k}}}\;{𝒨}_{\frac{n-\pi_{\mathrm{sub}}(k)}{2},\;\frac{n+1-\pi_{\mathrm{sub}}(k)}{2}}\;(\frac{n}{l_{k}}x),$$

a solution of integration in Equation (A18) may be obtained by using the following formula

(A21) $$\begin{array}{l} \int_{0}^{\infty }x^{v-1}\;e^{-\mathrm{bx}}\;{𝒨}_{\lambda_{1},\;u_{1}-\frac{1}{2}}\;({\mathrm{xa}}_{1})\ldots {𝒨}_{\lambda_{m},\;u_{m}-\frac{1}{2}}\;({\mathrm{xa}}_{m})dx=a_{1}^{u_{1}}\cdots a_{m}^{u_{m}}{\left(b+A\right)}^{-v-M}\;\Gamma (v+M)\times \\ F_{A}\left(v+M;u_{1}-\lambda_{1},\ldots ,u_{m}-\lambda_{m};2u_{1},\ldots ,2u_{m};\;\frac{a_{1}}{b_{1}+A},\ldots ,\frac{a_{m}}{b_{m}+A}\right), \end{array}$$

where *M* = *u*_1_+· · ·+*u_m_* and *A* = (1/2)(*a*_1_+· · ·+*a_m_*) are valid for ℜ(*M* + *v*) > 0, and $ℜ(b\pm \frac{1}{2}a_{1}\pm \cdots \pm \frac{1}{2}a_{n})>0$. Here 𝒨*_k,m_*(*x*) is a Whittaker function of the first kind Equation (7.622) in [25] and *F_A_*(*α : β*_1_, . . ., *β_m_*; *γ*_1_, . . ., *γ_m_* : *z*_1_, . . ., *z_m_*) is a hypergeometric function of several variables Equation (9.19) in [25]. After replacing the integration part of Equation (A18) by Equation (A21), it can easily be shown that for *m* dimensional SAR system with *n* ≥ *m*, the moments of the sample maximum eigenvalue is

(A22) $$\begin{array}{l} \;\;\;\;\;\;\;\;\;\;\;\;E(\lambda_{\max}^{s})=𝒮\sum_{i,j=1}^{m}{\left(-1\right)}^{i+j}\;\sum_{\underset{k\neq i\Lambda \pi_{\mathrm{sub}}(k)\neq j}{\pi_{\mathrm{sub}}\in S_{m}}}\text{sgn}\left(\pi_{\mathrm{sub}}\right)\\ \int_{0}^{\infty }\prod_{k=1}^{m-1}\;\frac{1}{n+1-\pi_{\mathrm{sub}}\;(k)}\;{\left(\frac{n}{l_{k}}\right)}^{\frac{n-\pi_{\mathrm{sub}}\;(k)}{2}}\;x^{s+n-i+\frac{n-\pi_{\mathrm{sub}}\;(k)}{2}}\;\text{exp}(-x(\frac{n}{l_{j}}+\frac{n}{2l_{k}}))\;{𝒨}_{\frac{n-\pi_{\mathrm{sub}}\;(k)}{2},\;\frac{n+1-\pi_{\mathrm{sub}}(k)}{2}}\;(x\frac{n}{l_{k}})dx\\ E(\lambda_{\max}^{s})=𝒮\sum_{i,j=1}^{m}{\left(-1\right)}^{i+j}\sum_{\pi_{\mathrm{sub}}\in S_{m}}\text{sgn}(\pi_{\mathrm{sub}})\prod_{k=1}^{m-1}\frac{1}{n+1-\pi_{\mathrm{sub}}\;(k)}\;{\left(\frac{n}{l_{k}}\right)}^{\frac{n-\pi_{\mathrm{sub}}\;(k)}{2}}\;a_{k}^{u_{k}}\times \\ \;\;\;\;\;\;\;\;\;\;\;\;{\left(b+A\right)}^{-v-M}\;\Gamma (v+M)\times F_{A}(v+M;u_{1}-\lambda_{1},\ldots ,u_{m-1}-\lambda_{m-1};2u_{1},\ldots ,2u_{m-1};\;\frac{a_{1}}{b_{1}+A},\\ \;\;\;\;\;\;\;\;\;\;\;\;\ldots ,\frac{a_{m-1}}{b_{m-1}+A}) \end{array}$$

where $a_{k}=\frac{n}{l_{k}}$, *u_k_* = (2+*n* − *π_sub_*(*k*))/2, $v=1+s+n-i+\sum_{k=1}^{m-1}\frac{n-\pi_{\mathrm{sub}}\;(k)}{2}$, $b=\frac{n}{l_{j}}+\sum_{k=1}^{m-1}\frac{n}{2l_{k}}$, $\lambda_{k}=\frac{n-\pi_{\mathrm{sub}}\;(k)}{2}$, $M=\sum_{k=1}^{m-1}u_{k}$ and $A=\frac{1}{2}\sum_{k=1}^{m-1}a_{k}$. Notice that *k* and *π_sub_*(*k*) takes values from (*k*, *π_sub_*(*k*) = {*k*, *π_sub_*(*k*) ∈ {1, 2, . . ., *m*}, *k* ≠ *j* ∧ *π_sub_*(*j*) ≠ *i*}) related to given *i*, *j*.

Although Equation (A22) is an exact closed-form expression for the MGF of the sample maximum eigenvalue, the hypergeometric function of several variables is in general very difficult to evaluate, and it is better to express Equation (A22) in a more tractable form for future analysis. Regarding Equation (2.53) in [21], the incomplete gamma function can be written in the closed form

(A23) $$\gamma (n+1-\pi_{\mathrm{sub}}\;(k),\;x\frac{n}{l_{k}})=(n-\pi_{\mathrm{sub}}\;(k))!\left(1-\text{exp}\left(-x\frac{n}{l_{k}}\right)\;\left(\sum_{t=0}^{n-\pi_{\mathrm{sub}}\;(k)}\frac{{\left(x\frac{n}{l_{k}}\right)}^{t}}{t!}\right)\right)$$

for *n* ∈ ℤ^+^, which is a constraint fulfilled by our problem. For the sake of simplicity, $f(k,\;\pi_{\mathrm{sub}(k)})=\text{exp}\left(-x\frac{n}{l_{k}}\right)\;\left(\sum_{t=0}^{n-\pi_{\mathrm{sub}}\;(k)}\frac{{\left(x\frac{n}{l_{k}}\right)}^{t}}{t!}\right)$, and $\prod_{k=1}^{m}(n-\pi_{\mathrm{sub}}\;(k))!\;(1-f(k,\;\pi_{\mathrm{sub}(k)}))=\prod_{k=1}^{m}a_{k}!(1-f_{k})$ indicate *m* times multiplications of incomplete gamma function. Then if this multiplication is extended as in the form

(A24) $$\prod_{k=1}^{m}a_{k}!\left(1+\sum_{k_{1}=1}^{\left(\begin{array}{c} m\\ 1 \end{array}\right)}{\left(-1\right)}^{1}f_{k_{1}}+\sum_{k_{2}=2>k_{1}=1}^{\left(\begin{array}{c} m\\ 2 \end{array}\right)}{\left(-1\right)}^{2}f_{k_{1}}f_{k_{2}}+\cdots +\sum_{k_{m}=m>\cdots >k_{2}=2>k_{1}=1}^{\left(\begin{array}{c} m\\ m \end{array}\right)}{\left(-1\right)}^{m}f_{k_{1}}f_{k_{2}}\cdots f_{k_{m}}\right)$$

and the integration can be easily implemented into Equation (A18) as follows

(A25) $$\begin{array}{r} E(\lambda_{\max}^{s})=𝒮\sum_{i,j=1}^{m}{\left(-1\right)}^{i+j}\sum_{\pi_{\mathrm{sub}}\in S_{m}}\text{sgn}(\pi_{\mathrm{sub}})\int_{0}^{\infty }x^{s+n-i}\;\text{exp}\left(-\frac{n}{l_{j}}x\right)(\prod_{k=1}^{m}(n-\pi_{\mathrm{sub}}(k))!\\ (1-f(k,\pi_{\mathrm{sub}(k)})))/\Psi_{\mathrm{ji}}\;dx \end{array}$$

By analyzing that the term Ψ*_ji_* in the determinant of Equation (A25) reduces to (*m* − 1) multiplication of incomplete gamma functions:

(A26) $$\begin{array}{l} E(\lambda_{\max}^{s})=𝒮\sum_{i,j=1}^{m}{\left(-1\right)}^{i+j}\;\sum_{\pi_{\mathrm{sub}}\in S_{m}}\text{sgn}(\pi_{\mathrm{sub}})\prod_{k=1}^{m}a_{k}!(\int_{0}^{\infty }x^{s}\;\text{exp}\left(-\frac{n}{l_{j}}x\right)\;x^{n-i}dx+\sum_{k_{1}=1}^{\left(\begin{array}{c} m-1\\ 1 \end{array}\right)}\int_{0}^{\infty }f_{k_{1}}\;x^{s}\\ \text{exp}\left(-\frac{n}{l_{j}}x\right)x^{n-i}\;dx+\cdots +\sum_{k_{m-1}=m-1>\cdots >k_{2}=2>k_{1}=1}^{\left(\begin{array}{c} m-1\\ m-1 \end{array}\right)}\int_{0}^{\infty }f_{k_{1}}\;f_{k_{2}}\cdots f_{k_{m-1}}\;x^{s}\;\text{exp}\left(-\frac{n}{l_{j}}x\right)x^{n-i}\;dx). \end{array}$$

Applying the rule $\int_{0}^{\infty }x^{n}\;e^{-ux}\;dx=n!u^{-n-1}$ in Equation (A26) it yields:

(A27) $$\begin{array}{ll} E(\lambda_{\max}^{s}) & =𝒮\sum_{i,j=1}^{m}{\left(-1\right)}^{i+j}\sum_{\pi_{\mathrm{sub}}\in S_{m}}\text{sgn}(\pi_{\mathrm{sub}})\prod_{k=1}^{m-1}a_{k}!\\ & \;\;\;\;\;(\frac{(n-i+s)!}{{\left(\frac{n}{l_{j}}\right)}^{n-i+s+1}}+\sum_{k_{1}=1}^{\left(\begin{array}{c} m-1\\ 1 \end{array}\right)}\sum_{t_{k_{1}}=0}^{n-\pi_{\mathrm{sub}}(k_{1})}\frac{{\left(\frac{n}{l_{k_{1}}}\right)}^{t_{k_{1}}}}{t_{k_{1}}!}\frac{(s+n-i+t_{k_{1}})!}{{\left(\frac{n}{l_{j}}+\frac{n}{l_{k_{1}}}\right)}^{s+n-i+t_{k_{1}}+1}}+\cdots +\\ & \;\;\;\;\;\sum_{t_{k_{m-1}}=0}^{n-\pi_{\mathrm{sub}}(k_{m-1})}\frac{{\left(\frac{n}{l_{k_{m-1}}}\right)}^{t_{k_{m-1}}}}{t_{k_{m-1}}!}\cdots \sum_{t_{k_{1}}=0}^{n-\pi_{\mathrm{sub}}(k_{1})}\frac{{\left(\frac{n}{l_{k_{1}}}\right)}^{t_{k_{1}}}}{t_{k_{1}}!}\frac{(s+n-i+t_{k_{1}}+\cdots +t_{k_{m-1}})!}{{\left(\frac{n}{l_{j}}+\frac{n}{l_{k_{1}}}+\cdots +\frac{n}{l_{k_{m-1}}}\right)}^{s+n-i+t_{k_{1}}+\cdots +t_{k_{m-1}}+1}}). \end{array}$$

The *s*th moment of sample maximum eigenvalue can finally be written in a closed-form expression as

(A28) $$E(\lambda_{\max\;}^{s})=𝒮\sum_{i,j=1}^{m}\sum_{\pi_{\mathrm{sub}}\in S_{m}}\text{sgn}(\pi_{\mathrm{sub}})𝒢(k,\pi_{\mathrm{sub}}(k)),\;\text{if }k=j\wedge \pi_{k}(j)=i,\;\pi_{k}\in \pi_{\mathrm{sub}}$$

(A29) $$\begin{array}{lll} 𝒢(k,\;\pi_{\mathrm{sub}}\;(k)) & = & \prod_{k=1}^{m-1}(n-\pi_{\mathrm{sub}}\;(k))!\times (\frac{(n-i+s)!}{{\left(\frac{n}{l_{j}}\right)}^{n-i+s+1}}\\ & + & \sum_{k_{1}=1}^{\left(\begin{array}{c} m-1\\ 1 \end{array}\right)}\sum_{t_{k_{1}}=0}^{n-\pi_{\mathrm{sub}}(k_{1})}\frac{{\left(\frac{n}{l_{k_{1}}}\right)}^{t_{k_{1}}}}{t_{k_{1}}!}\frac{(s+n-i+t_{k_{1}})!}{{\left(\frac{n}{l_{j}}+\frac{n}{l_{k_{1}}}\right)}^{s+n-i+t_{k_{1}}+1}}\\ & + & \sum_{k_{2}=2>k_{1}=1}^{\left(\begin{array}{c} m-1\\ 2 \end{array}\right)}\sum_{t_{k_{1}}=0}^{n-\pi_{\mathrm{sub}}(k_{1})}\frac{{\left(\frac{n}{l_{k_{1}}}\right)}^{t_{k_{1}}}}{t_{k_{1}}!}\sum_{t_{k_{2}}=0}^{n-\pi_{\mathrm{sub}}(k_{2})}\frac{{\left(\frac{n}{l_{k_{2}}}\right)}^{t_{k_{2}}}}{t_{k_{2}}!}\frac{(s+n-i+t_{k_{1}}+t_{k_{2}})!}{{\left(\frac{n}{l_{j}}+\frac{n}{l_{k_{1}}}+\frac{n}{l_{k_{2}}}\right)}^{s+n-i+t_{k_{1}}+t_{k_{2}}+1}}\\ & + & \cdots \\ & + & \sum_{t_{k_{m-1}=0}}^{n-\pi_{\mathrm{sub}}(k_{m-1})}\frac{{\left(\frac{n}{l_{k_{m-1}}}\right)}^{t_{k_{m-1}}}}{t_{k_{m-1}}!}\cdots \sum_{t_{k_{1}}=0}^{n-\pi_{\mathrm{sub}}(k_{1})}\frac{{\left(\frac{n}{l_{k_{1}}}\right)}^{t_{k_{1}}}}{t_{k_{1}}!} \end{array}$$

(A30) $$\frac{(s+n-i+t_{k_{1}}+\cdots +t_{k_{m-1}})!}{{\left(\frac{n}{l_{j}}+\frac{n}{l_{k_{1}}}+\cdots +\frac{n}{l_{k_{m-1}}}\right)}^{s+n-i+t_{k_{1}}+\cdots +t_{k_{m-1}}+1}})$$

The moments of the sample maximum eigenvalue regarding its PDF is solved. For larger *m* dimensional systems, such an approach may become complicated due to the need for a large number of calculation of cofactors. However, for applications where only a few eigenvalues, like polarimetry (*m* = 3) and interferometry (*m* = 2), are of interest, the numerical calculations are quite rapid and stable. For example, in the simplest case of *m* = 2, *n* ≥ 2, the MGF is given by

(A31) $$\begin{array}{ll} E(\lambda_{\max}^{s}) & =𝒮\left(\sum_{j,i=1}^{2}\int_{0}^{\infty }x^{s}x^{n-i}\;\text{exp}(-x\frac{n}{l_{j}})\sum_{\pi_{\mathrm{sub}}\in S_{m}}\text{sgn}(\pi_{\mathrm{sub}})\;\gamma (n+1-k,x\frac{n}{l_{\pi_{\mathrm{sub}}}(k)}){\left(\frac{n}{l_{\pi_{\mathrm{sub}}}(k)}\right)}^{k-n-1}\right)\\ & \text{if}\;\pi_{k}(j)=i,\;\;\pi_{k}\in \pi_{\mathrm{sub}},\;\;\;\;\text{and}\;\;\;\text{if}\;k\neq j\;\;\text{and}\;\;l_{\pi_{\mathrm{sub}}(k)}\neq i,\;k=1,\;2\\ & =𝒮\Gamma (2n)\Gamma (n-1){\left(\frac{l_{1}l_{2}}{nl_{1}+nl_{2}}\right)}^{2n}\\ & \times \left(\sum_{j,i=1}^{2}\sum_{\pi_{\mathrm{sub}}\in S_{m}}\text{sgn}(\pi_{\mathrm{sub}}){\left(n+1-k\right)}_{2}F_{1}(1,2n;n+2-k;\frac{l_{i}}{l_{i}+l_{\pi_{\mathrm{sub}}(k)}})\right). \end{array}$$

where the constant $𝒮=\frac{{\left(l_{1}l_{2}\right)}^{1-n}n^{2n}}{\Gamma (n-1)\Gamma (n+1)(l_{1}-l_{2})}$ for *m* = 2 and _2_*F*_1_(*a*, *b; c; x*) is a Hypergeometric function Equation (9.8) in [21].
